# Finding phenazine

**DOI:** 10.7554/eLife.62983

**Published:** 2020-10-27

**Authors:** Sarah J Wolfson, Libusha Kelly

**Affiliations:** 1 Department of Systems and Computational Biology, Albert Einstein College of MedicineBronxUnited States; 2 Department of Microbiology and Immunology, Albert Einstein College of MedicineBronxUnited States

**Keywords:** phenazines, metagenomics, ecology, Other

## Abstract

Analysis of genetic information from soil samples provides insights into bacteria that help to protect crops from fungal diseases by producing chemicals called phenazines.

**Related research article** Dar D, Thomashow LS, Weller DM, Newman DK. 2020. Global landscape of phenazine biosynthesis and biodegradation reveals species-specific colonization patterns in agricultural soils and crop microbiomes. *eLife* **9**:e59726. doi: 10.7554/eLife.59726

“Imagine walking out in the countryside and not being able to tell a snake from a cow from a mouse from a blade of grass” (Carl Woese, in Yardley, 2012). A similar problem confronted researchers trying to identify individual microbes within complex communities – ecosystems that can contain thousands of different microbial species – before DNA sequencing made it easier to distinguish different microorganisms (Woese and Fox, 1977). Part of the problem was that microbes are wildly diverse, spanning the three domains of life. Moreover, two microbes can be as dissimilar as mushrooms and humans, yet difficult to tell apart – even with the help of a microscope.

Complex microbial communities are particularly important in agriculture. Most of the food crops we grow have been meticulously selected and modified to increase yields, among other things, but our understanding of how these crops interact with the wild microbes that live in soil is far from complete (Graham et al., 2016; Badri et al., 2009). Now, in eLife, Dianne Newman (California Institute of Technology) and colleagues – including Daniel Dar (CalTech), Linda Thomashow and David Weller (both from the USDA Agricultural Research Service) – report how studying metagenomes can shed light on the bacteria responsible for making phenazines, a class of chemicals that protects major food crops from fungal disease (Dar et al., 2020).

Until now, identifying the bacteria that produced phenazines was a slow process that relied on analyzing individual bacteria from different plant samples independently, or on comparing samples with mixed DNA and reporting on the relative proportions of bacteria (Mavrodi et al., 2013). Moreover, it was difficult to compare different samples using these methods. The new metagenomic technique – which involves analyzing genetic material collected from soil samples – does not suffer from these shortcomings.

Dar et al. started by connecting specific bacteria found in the immediate vicinity of plant roots – a region of soil called the rhizosphere – to the production of phenazine. They searched for the genes that allow bacteria to make phenazine in agricultural soil samples, and assumed that any bacteria carrying these genes were indeed true phenazine producers. However, simply counting the number of these 'phz+' bacteria in each sample was not sufficient as no two grams of soil contain the same number of bacteria. Dar et al. allowed for this by dividing the number of phz+ bacteria by the number of individual bacteria in each sample (which can be estimated by counting certain genes that are found in all bacteria in single copy; Parks et al., 2018). The value of this ratio can be compared across multiple samples from different environments.

After confirming that their pipeline worked by testing it on computationally-generated data, Dar et al. applied their approach to a real meta- genomic dataset from the rhizosphere of wheat. This revealed that phenazines were produced by two groups of bacteria. The bacteria in one of these groups belong to the *Pseudomonas* genus, and were already known to produce phenazine based on traditional culture based studies. However, the discovery of a second group, *Streptomyces* bacteria, came as a surprise as there are no previous reports of any members of this diverse group of bacteria being phenazine producers relevant to agricultural crops. This discovery is agriculturally relevant because different bacteria can produce different phenazine compounds, which interact with roots in different ways.

Based on these results, Dar et al. expanded their search to 799 more datasets and found that phz+ bacteria comprised between 0% and 2.7% of the total bacteria in the samples. Some crops harbor more phz+ bacteria than others and, on average, the rhizosphere contained 1.9 times more phz+ bacteria than 'open' soil. Some strains of phz+ bacteria were also plant-specific, while others inhabited the rhizospheres of multiple plants as well as open soils (Figure 1). The new analysis also provided insights into what bacterial species are important phenazine producers. The phz+ *Streptomyces* detected initially comprised a large portion of phenazine producers. In addition, a clade of bacteria previously unknown to colonize major crops, Xanthomonadales, was often found associated with root ecosystems highly enriched in phz+ bacteria.

**Figure 1.**
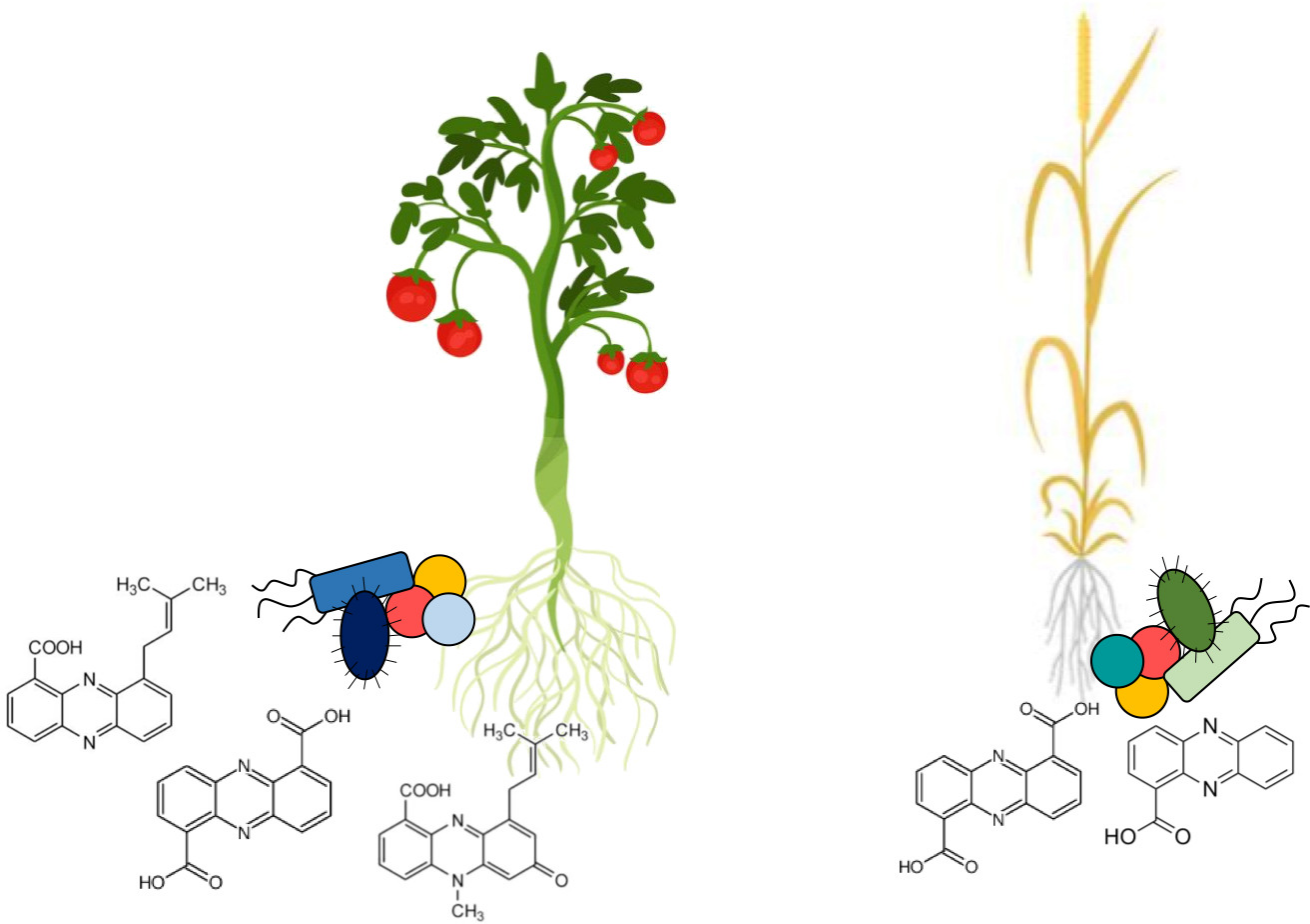
The rhizomes of different food crops host distinct communities of phenazine-producing bacteria. Root microbiomes are enriched in bacteria that produce phenazines, a group of compounds that protects plants from fungal diseases. Dar et al. have developed a method that allows them to compare which phenazine-producing bacteria are present in different soil and crop samples. This allows them to identify which phenazine-producing bacteria may be important for different food crops. Some of the phenazine-producing microbes are associated with specific plants: on the left, tomatoes are shown with specific microbes in blue, while on the right wheat is shown with specific microbes in green. Other microbes are common across different ecosystems: yellow and peach-colored coccoid microbes are shown with both crops. Under each crop, the chemical structures of different types of phenazines show the diversity of these compounds, which depends on the bacteria producing them.

Finally, to confirm that the analysis could identify genes within phz+ bacteria that produce specific phenazines, Dar et al. cultured different genetically modified versions of one Xanthomonadales species in the laboratory. When the genes predicted to be involved in phenazine production were removed from the different versions of this bacterium, the bacteria stopped producing phenazine.

Researchers currently have access to a wide range of metagenomic datasets, and the approach developed by Dar et al. provides new ways to analyze these and find out more about the interactions between bacterial microbes and plants. Normalizing bacterial counts across samples allows scientists to uncover global microbial interactions, and potentially predict the chemical environment of an ecosystem. The ability of microbes to shape their own chemical environment is an emerging area of research (Guthrie et al., 2019; Hooper et al., 2002; Reese et al., 2018) that is likely relevant across many microbiomes, from the soil to the human body.
